# Androgen deficiency in male patients diagnosed with ANCA-associated vasculitis: a cause of fatigue and reduced health-related quality of life?

**DOI:** 10.1186/ar4297

**Published:** 2013-09-12

**Authors:** Janneke Tuin, Jan-Stephan F Sanders, Birgit M Buhl, André P van Beek, Coen A Stegeman

**Affiliations:** 1 Department of Internal Medicine, Division of Nephrology, University of Groningen, University Medical Center Groningen, The Netherlands; 2 Department of Internal Medicine, Division of Rheumatology, University of Groningen, University Medical Center Groningen, The Netherlands; 3 Department of Internal Medicine, Division of Endocrinology, University of Groningen, University Medical Center Groningen, The Netherlands

**Keywords:** Fatigue, health-related quality of life (HRQOL), physical functioning, testosterone, androgen deficiency, ANCA-associated vasculitis

## Abstract

**Introduction:**

Low testosterone levels in men are associated with fatigue, limited physical performance and reduced health-related quality of life (HRQOL); however, this relationship has never been assessed in patients with anti-neutrophil cytoplasmic antibodies (ANCA) -associated vasculitides (AAV). The aim of this study was to assess the prevalence of androgen deficiency and to investigate the role of testosterone in fatigue, limited physical condition and reduced HRQOL in men with AAV.

**Methods:**

Male patients with AAV in remission were included in this study. Fatigue and HRQOL were assessed by the multi-dimensional fatigue inventory (MFI)-20 and RAND-36 questionnaires.

**Results:**

Seventy male patients with a mean age of 59 years (SD 12) were included. Scores of almost all subscales of both questionnaires were significantly worse in patients compared to controls. Mean total testosterone and free testosterone levels were 13.8 nmol/L (SD 5.6) and 256 pmol/L (SD 102), respectively. Androgen deficiency (defined according to Endocrine Society Clinical Practice Guidelines) was present in 47% of patients. Scores in the subscales of general health perception, physical functioning and reduced activity were significantly worse in patients with androgen deficiency compared to patients with normal androgen levels. Testosterone and age were predictors for the RAND-36 physical component summary in multiple linear regression analysis. Testosterone, age, vasculitis damage index (VDI) and C-reactive protein (CRP) were associated with the MFI-20 subscale of general fatigue.

**Conclusions:**

This study showed that androgen deficiency was present in a substantial number of patients with AAV. Testosterone was one of the predictors for physical functioning and fatigue. Testosterone may play a role in fatigue, reduced physical performance and HRQOL in male patients with AAV.

## Introduction

Granulomatosis with polyangiitis (GPA), microscopic polyangiitis (MPA) and eosinophilic granulomatosis with polyangiitis (EGPA) are associated with anti-neutrophil cytoplasmic antibodies (ANCA) and, therefore, are also known as ANCA-associated vasculitides (AAV). This group of autoimmune diseases is characterized by inflammation of the small to medium-sized blood vessels, which can affect a variety of organs [1]. The upper airways, lungs and kidneys are most frequently involved and this may lead to organ failure with potentially life-threatening outcome. The combination of cyclophosphamide (CY) and high dose glucocorticoids (GC) forms the cornerstone of induction therapy. With this therapy life expectancy has increased significantly with current five-year survival rates exceeding 80% [2]. Consequently, these previously fatal diseases have evolved into chronic relapsing illnesses.

Fatigue and reduced physical condition are frequently expressed complaints by patients, even after attainment of remission. In addition, several studies have demonstrated that many patients with these diseases experience a reduced health-related quality of life (HRQOL) and impairments in daily life, work and social activities[3,4]. Since these diseases have become chronic diseases, more attention has been payed to the assessment of HRQOL and its contributing factors. Pain, presence of neuropathy, glucocorticoid treatment, neurologic involvement at diagnosis and employement status are considered to influence HRQOL [4-6]. Fatigue is also thought to be a major contributor to impaired quality of life [7].

Low testosterone levels are known to be associated with fatigue and diminished performance. Previously, a small study reported the prevalence of hypogonadism in 19 male patients with GPA [8]. More than 50% of patients fulfilled the proposed criteria of hypogonadism in that study group; however, the relationship between testosterone and fatigue was not assessed.

The aim of the current study was to investigate the prevalence and determinants of androgen deficiency in a large group of men with AAV in stable remission and the possible relation between testosterone levels and physical performance.

## Methods

### Patients

Male patients diagnosed with GPA, MPA or EGPA in stable remission and visiting the outpatient clinic of the University Medical Center Groningen (UMCG) between October 2010 and October 2011 were asked to participate in this cross-sectional study. Patients were eligible if therapy with cyclophosphamide had been stopped at least six months prior to study entry. Patients were classified as GPA, MPA or EGPA according to criteria adopted from the Chapel Hill Consensus Conference Nomenclature and the algorithm developed by Watts *et al*. [1,9]. Data were collected on patient, disease and treatment characteristics through medical records. Informed consent was obtained at the visit to the outpatient clinic. The study was approved by the Medical Ethical Board of the University Medical Center Groningen as part of a larger study on biomarkers and disease activity and damage in ANCA-associated vasculitis (METc number 2010/057).

### Clinical assessment and questionnaires

Disease activity at time of assessment was scored using the Birmingham Vasculitis Activity Score (BVAS) [10]. Stable remission was defined as BVAS 0 and no signs or symptoms of disease activity. Chronic damage due to disease, treatment or unrelated events was scored by the Vasculitis Damage Index (VDI) at the time of study [11].

For the assessment of HRQOL the RAND- 36 health survey was used. In this self-report questionnaire HRQOL is divided into eight subscales: general health perception, physical functioning, social functioning, role limitations due to physical problems, role limitations due to emotional problems, pain, vitality and mental health. Normative data were derived from a screening of 1,063 persons in the municipality of Emmen in The Netherlands. Scores in every subscale range from 0 to 100, with a higher score reflecting a higher level of functioning [12]. The eight domains of the RAND-36 were summarized resulting in the physical component summary (PCS) and the mental component summary (MCS), with a population mean of 50 SD 10 [13]. For the assessment of fatigue the Multi-dimensional Fatigue Inventory (MFI-20) was used [14]. This self-report questionnaire contains 20-items, covering five dimensions of fatigue: general fatigue, physical fatigue, reduced activity, reduced motivation and mental fatigue. Scores in every dimension range from 4 to 20, a higher score corresponding to a higher level of fatigue. The subscale of general fatigue was considered as the overall fatigue score encompassing both physical and mental aspects of fatigue, as proposed by the authors [14]. Normative data were derived from a healthy Dutch population described by Smets *et al*. [15]. Both questionnaires were in Dutch. The study cohort and reference population were not matched.

### Laboratory parameters

All laboratory parameters were measured at the time of the assessment. Creatinine clearance (CC) was obtained from 24-hour urine collection; if not available, CC was estimated using the Cockcroft-Gault formula [16]. Normal kidney function was defined as CC of >90 ml/minute together with proteinuria of <0.3 g/24 hours.

Total testosterone was analyzed by radioimmunoassay, using (1,2,6,73H)-testosterone as tracer (Amersham Biosciences, Buckinghamshire, UK) [17]. The coefficient of the intra-assay variation was 4.5%, and of the inter-assay variation was 10.5%. Sex hormone-binding globulin (SHBG) was measured by in-house radioimmunoassay, using a binding assay. Hemoglobin and albumin were obtained from routine blood investigations. SHBG and albumin were measured to calculate the free testosterone from total testosterone according to the formula by Vermeulen [18].

### Definitions

Male patients were considered to have androgen deficiency when the level of total testosterone was <10 nmol/L or 10 to 12 nmol/L in combination with free testosterone <0.30 nmol/L (lower limit UMCG), as recommended by the Endocrine Society Clinical Practice Guidelines [19].

### Statistical analyses

Data were analyzed using SPSS 20.0. Graphs were made with GraphPad Prism softare (version 5.0) and SigmaPlot (version 11.0). Mean plus standard deviation (SD) and median plus interquartile range (IQR) were used for summary data. For the comparisons of normally distributed data the Student's t-test was used. Not normally distributed data were compared using the Mann-Whitney U-test. If indicated, not normally distributed data were log transformed. Z-scores were calculated for comparison of the scores of the RAND-36 and the MFI-20 between patients with androgen deficiency and normal androgen levels. Backward stepwise multiple linear regression analyses were performed on PCS, MCS and the subscale of general fatigue. The following candidate predictors were included: total testosterone; age; interaction between age and testosterone; hemoglobin; CC; C-reactive protein (CRP); VDI; disease duration; log-transformed cumulative GC dose and log-transformed cumulative CY dose. Backward stepwise multiple linear regression analysis was also performed on the level of total testosterone. The following candidate predictors were included: age; SHBG; VDI; body mass index (BMI); hemoglobin; current glucocorticoid use; CRP; log-transformed cumulative cyclophosphamide dose and the log-transformed cumulative glucocorticoid dose. Statistical significance was defined as a two-sided *P *≤ 0.05.

## Results

### Patient characteristics at diagnosis and study

Seventy male patients diagnosed with GPA (*n *= 54), MPA (*n *= 14) and EGPA (*n *= 2) between 1984 and 2009 were included. The mean age at diagnosis was 50 years (SD 14). In five patients the disease was limited to the ear, nose and throat (ENT) region, eyes, orbita or a combination; in all others generalized vasculitis had been present during the course of the disease. The ENT region (63%), kidneys (56%) and lungs (51%) were most frequently involved. ANCA against proteinase 3 (PR-3) were present in 50 patients, ANCA against myeloperoxidase (MPO) were present in 18 patients and ANCA without specificity were present in two patients. Sixty-seven patients had been treated with cyclophosphamide, two patients with rituximab and one had been treated only with co-trimoxazole monotherapy prior to this study. One of the included patients developed a transient reduction of total testosterone at diagnosis. A second patient experienced a testicular infarction with extensive hemorrhage and inflammation resulting in a one-sided orchidectomy. A third patient had involvement of the testes at diagnosis. Testosterone levels in all three patients had returned or were proven to be within the normal range after induction of remission in a period prior to this study. None of the included patients was or had been treated with androgen substitution therapy.

Forty-three patients experienced one or more relapse(s) and 27 patients had no relapse before inclusion in the current study. An overview of patient and laboratory characteristics at the time of study is presented in Table 1.

**Table 1:** Patient and laboratory characteristics at time of study

Characteristics	All patients (number = 70)
Age at study (years), mean (SD)	59 (12)
Diagnosis to study (months), median (IQR)	79 (44 to 149)
Last relapse to study (months), median (IQR)	39 (19 to 68)
BVAS = 0, n	70
VDI, median (IQR)	3 (2 to 4)
Cum. CY (g.), median (IQR)	27.5 (17.2 to 57.8)
Cum. GC (g.), median (IQR)	15.3 (7.0 to 26.0)
TT (nmol/L), mean (SD)	13.8 (5.6)
FT (pmol/L), mean (SD)	256 (102)
SHBG (nmol/L), mean (SD)	39 (14)
Hb (mmol/L), mean (SD)	8.7 (1.0)
CRP (mg/L), median (IQR)	<5 (<5 to 6)
CC (ml/min), mean (SD)	90 (30)

BVAS, Birmingham Vasculitis Activity Score; CC, creatinine clearance; CRP, C-reactive protein; Cum. CY, cumulative cyclophosphamide; Cum. GC, cumulative glucocorticoid; FT, free testosterone; Hb, hemoglobin; SHBG, sex hormone-binding globulin; TT, total testosterone; VDI, Vasculitis Damage Index.

### Androgen status

**Figure 1:**
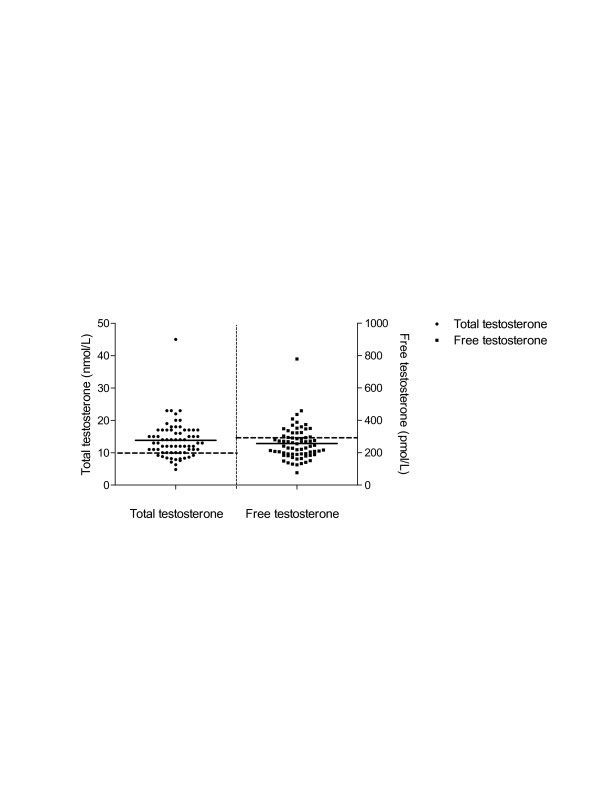
**Mean total and free testosterone**. Normal values are represented by the dashed lines, respectively, at 10 nmol/L for total testosterone and 300 pmol/L for free testosterone.

Mean total and free testosterone levels were 13.8 nmol/L (SD 5.6) and 256 pmol/L (SD 102), respectively (Figure 1). Thirty-three patients (47%) had androgen deficiency according to the definition of the Endocrine Society Clinical Practice Guidelines [19]. Seven percent of patients under 50 years of age (*n *= 14) and 57% of patients over 50 years of age (*n *= 56) had androgen deficiency. The mean level of SHBG was 39 nmol/L (SD 14), and 51 patients (73%) had SHBG levels above the upper reference of our center, 30 nmol/L. No known SHBG-increasing conditions, for example, hepatic cirrhosis, hepatitis, hyperthyroidism, use of anticonvulsants or HIV, were present in any of our patients [19]. The use of glucocorticoids at the time of the assessment was significantly associated with lower levels of SHBG, a known effect of glucocorticoid use.

### Scores on the RAND-36 and MFI-20

**Figure 2:**
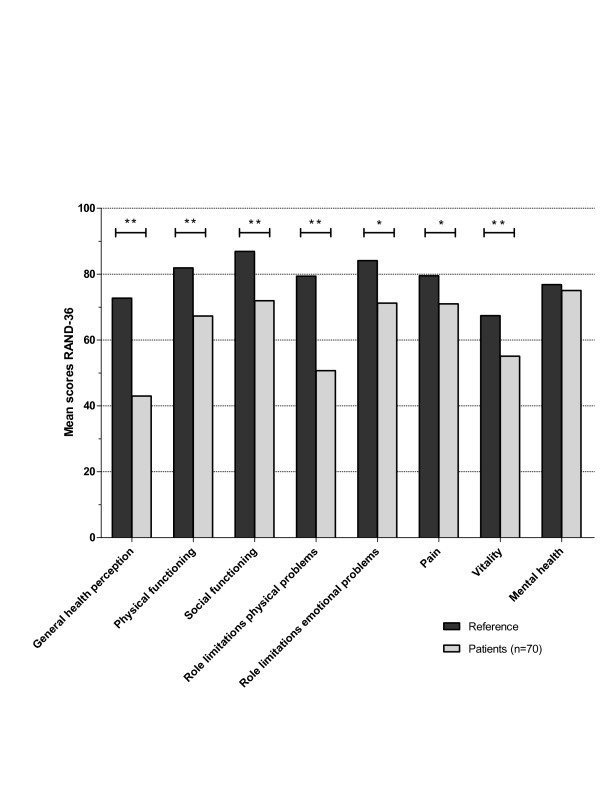
**Mean scores in the subscales of the RAND-36 of all patients and references**. A *P *value of *P *< 0.010 is shown as * and *P *< 0.001 as **.

Scores in every subscale of the RAND-36, except for the subscale of mental health, were significantly lower in patients with AAV compared to the control population (*P *values ranged from < 0.001 to 0.009) (see Figure 2). The mean PCS score was 41.4 (SD 11.4) which is significantly lower than in the mean population (*P *< 0.001). The mean MCS score was 47.8 (SD 9.5) which is not significantly lower than that of the mean population (*P *= 0.055).

**Figure 3:**
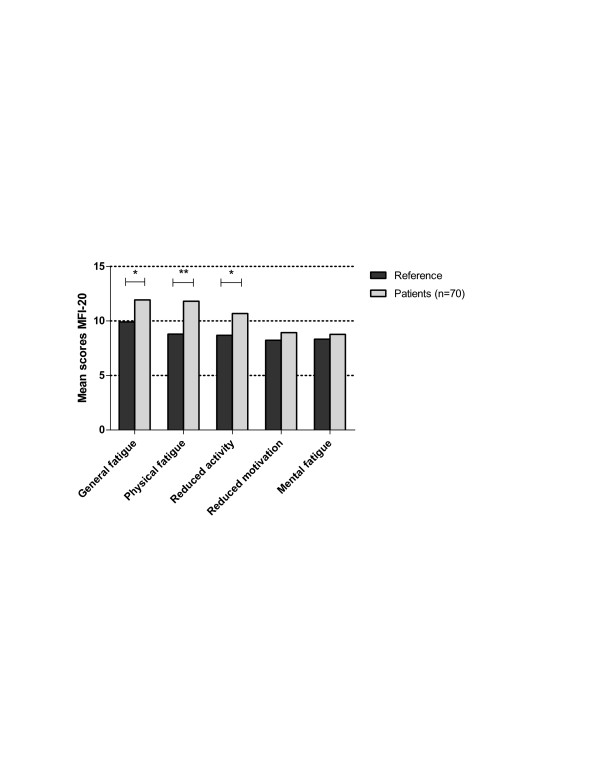
**Mean scores in the subscales of the MFI-20 of all patients and references**. A *P *value of *P *< 0.010 is shown as * and *P *< 0.001 as **.

Scores in the subscales of general fatigue (*P *= 0.001), physical fatigue (*P *< 0.001) and reduced activity (*P *= 0.001) of the MFI-20 were significantly higher compared to the reference scores. The subscales of reduced motivation and mental fatigue were not significantly higher (see Figure 3).

**Figure 4:**
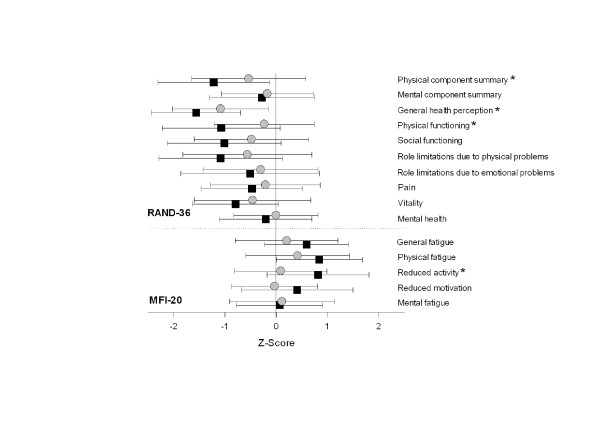
**Z-scores of the RAND-36 and MFI-20 questionnaires**. Grey circles represent mean and SD of patients with normal androgen levels (*n *= 37) and black squares represent mean and SD of patients with androgen deficiency (*n *= 33). Mean population score is represented by a Z-score of zero. *P *< 0.05 is indicated with *. Note that higher Z-scores on the MFI-20 mean more fatigue and lower Z-scores on the RAND-36 signify poorer quality of life.

Z-scores of the PCS, subscale of general health perception and subscale of physical functioning (RAND-36) were significantly lower and scores in the subscale of reduced activity (MFI-20) were significantly higher in patients with androgen deficiency compared to patients with normal androgen levels. In addition, scores in the subscales of social functioning (*P *= 0.052), role limitations due to physical problems (*P *= 0.081), general fatigue (*P *= 0.081), physical fatigue (*P *= 0.062) and reduced motivation (*P *= 0.064) tended to be worse in patients with androgen deficiency (see Figure 4). The patient characteristics for patients with (*n *= 33) and without (*n *= 37) androgen deficiency are presented in Table 2. Additional file 1, Table S1 shows a broad overview of scores of the RAND-36 and MFI-20 questionnaires.

**Table 2:** Patient, treatment and laboratory characteristics of patients with androgen deficiency and patients with normal androgen levels

Characteristics	Normal androgen levels (number = 37)	Androgen deficiency (number = 33)	*P*
Age at study (years), mean (SD)	55 (13)	64 (10)	0.005
Diagnosis to study (months), median (IQR)	75 (39 to 119)	129 (70 to 168)	0.041
Last relapse to study (months), median (IQR)	36 (19 to 67)	49 (19 to 73)	0.403
BVAS = 0, n	37	33	
VDI, median (IQR)	3 (2 to 4)	4 (2 to 5)	0.045
Cum. CY (g.), median (IQR)	22.4 (15.5 to 39.8)	46.6 (18.0 to 92.0)	0.049
Cum. GC (g.), median (IQR)	10.6 (6.1 to 18.4)	21.7 (13.5 to 33.0)	0.001
TT (nmol/L), mean (SD)	17.4 (5.5)	9.8 (1.9)	<0.001
FT (pmol/L), mean (SD)	313 (103)	193 (51)	<0.001
SHBG (nmol/L), mean (SD)	43 (12)	35 (15)	0.029
Hb (mmol/L), mean (SD)	9.0 (1.0)	8.4 (0.9)	0.009
CRP (mg/L), median (IQR)	<5 (<5 to <5)	<5 (<5 to 9)	0.072
CC (ml/min), mean (SD)	91 (27)	89 (39)	0.811
Treatment at study			
No treatment, number	11	7	
CT, number	9	5	
GC (+ CT), number	3 (1)	7 (2)	
Aza (+CT), number	8 (6)	2 (1)	
MMF (+CT), number	3 (3)	2 (1)	
GC and Aza (+CT), number	1 (1)	6 (6)	
GC and MMF (+CT), number	2 (2)	4 (3)	

Aza, azathioprine; BVAS, Birmingham Vasculitis Activity Score; CC, creatinine clearance; CRP, C-reactive protein; CT, co-trimoxazole, 2 dd 800/160 mg; Cum. CY, cumulative cyclophosphamide; Cum. GC, cumulative glucocorticoid; FT, free testosterone; Hb, hemoglobin; MMF, mycophenolate mofetil; SHBG, sex hormone-binding globulin; TT, total testosterone; VDI, Vasculitis Damage Index.

**Figure 5:**
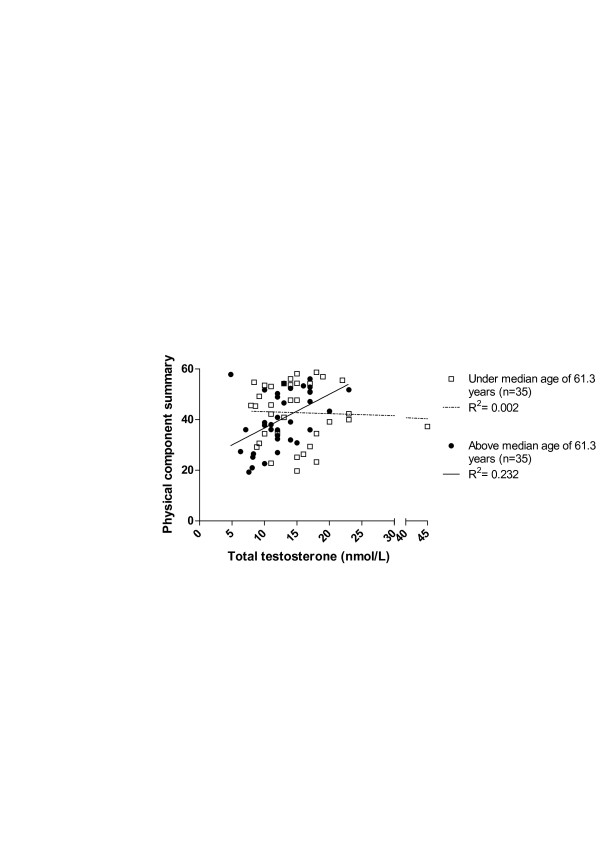
**Association between the physical component score and levels of total testosterone for patients under and above the median age of 61.3 years**. Open squares represent total testosterone levels and PCS scores of patients under the median age and the blue circles represent total testosterone levels and PCS scores of patients above the median age. PCS, physical component summary.

The predictors for physical functioning (PCS), mental functioning (MCS) and fatigue (subscale of general fatigue) were assessed in multiple linear regression analyses. Testosterone, age and the interaction between age and testosterone were predictors for the PCS (see Table 3). The subscale of general fatigue was associated with testosterone, age, the interaction between age and testosterone, CRP and VDI (see Table 4). None of the candidate predictors was significantly associated with the MCS. Figure 5 demonstrates the nature of the interaction between age and testosterone levels on PCS.

**Table 3:** Multiple linear regression model predicting for PCS

PCS	B (95% CI)	*P *value
Age, years	-0.917 (-1.569; -0.266)	0.007
Total testosterone, nmol/L	-2.837 (-5.293; -0.381)	0.024
Interaction age and total testosterone	0.57 (0.013; 0.102)	0.013

Adjusted R square 0.092; intercept 89.32 (95% CI, 51.572; 127.071). PCS, physical component summary.

**Table 4:** Multiple linear regression model predicting for general fatigue

General fatigue	B (95% CI)	*P *value
Age, years	0.301 (0.026; 0.575)	0.032
Total testosterone, nmol/L	1.045 (0.017; 2.073)	0.046
Interaction age and total testosterone	-0.019 (-0.038; 0.000)	0.045
VDI	0.642 (0.061; 1.223)	0.031
CRP	-0.123 (-0.238; -0.008)	0.037

Adjusted R square; 0.117 intercept -6.523 (95% CI, -22.426; 9.380). CRP, C-reactive protein; VDI, Vasculitis Damage Index.

### Determinants of levels of testosterone

Patients with androgen deficiency had a significantly longer disease duration, higher VDI scores and higher cumulative cyclophosphamide doses compared to patients with normal androgen levels, reflecting a higher disease burden and more immunosuppressive therapy (Table 2).

In multiple linear regression analysis total testosterone was significantly associated with the predictors age, SHBG and the log transformed cumulative glucocorticoid dose (Table 5).

**Table 5:** Multiple linear regression model predicting for level of total testosterone

	B (95% CI)	*P *value
Age, years	-0,15 (-0.25; -0.06)	0.002
SHBG, nmol/L	0,16 (0.07; 0.24)	0.000
Ln cum GC	-1,89 (-3.38; -0.40)	0.014

Adjusted R square 0.302; intercept 34.92 (95% CI, 19.16; 50.68). Ln cum GC, log-transformed cumulative glucocorticoid dose; SHBG, sex hormone-binding globulin;

## Discussion

Fatigue and limited physical condition are frequently expressed complaints by patients with AAV. Our data confirm the finding that AAV patients show considerably reduced HRQOL and increased fatigue. Several studies identified contributing factors to reduced HRQOL and fatigue; however, the role of sex hormones has not been studied. Here, we showed that there was a high prevalence of androgen deficiency among male patients with AAV and that testosterone levels were associated with physical functioning and fatigue in multiple linear regression analyses. Furthermore, we found indications that longer duration of disease and treatment, especially long-term use of glucocorticoids, was associated with reduced androgen concentrations in male patients with AAV.

Androgen deficiency was more prevalent in this study (47%) compared to the normal population [20]. Aging is associated with a decline of testosterone levels; however, this physiological decline is rather subtle and is estimated to be -0.4% to -0.8% per year in cross-sectional studies [21,22]. Patients with androgen deficiency were significantly older compared to patients with normal androgen levels, and, therefore, an age-related decline of a few percent was expected. However, testosterone levels in patients with androgen deficiency were more than 40% lower.

Besides age and SHBG, the cumulative glucocorticoid dose was associated with the level of total testosterone, suggesting a role for treatment with glucocorticoids in this decline. It is unknown whether glucocorticoids themselves, as toxic drugs, reduce androgen production or whether a higher cumulative dose reflects severe disease, which may be associated with reduced testosterone production. Previous research showed that chronic glucocorticoid therapy can result in a decrease of testosterone [23-26]. In addition, disease related damage reflected by the VDI, disease duration and the cumulative cyclophosphamide dose were significantly higher in patients with androgen deficiency. These variables, especially the cumulative cyclophosphamide dose, were not significantly associated with levels of testosterone in multiple linear regression analyses. Over time, the cumulative cyclophosphamide dose has been reduced; therefore, a possible relation between cyclophosphamide and testosterone might be difficult to demonstrate.

Our findings are in line with the findings of Richter *et al*. who reported a high prevalence of hypogonadism in a small cohort of male patients with AAV [8]. In contrast to Richter *et al*., we found indications that the cumulative glucocorticoid doses were associated with levels of testosterone. It is not clear what caused the opposite finding; however, our cohort consisted of more patients, patients had a longer disease duration and twice as high median cumulative glucocorticoid doses. To our knowledge, Richter *et al*. were the first to report data on sex hormones in a cohort of men with AAV; in patients with rheumatoid arthritis (RA) and systemic lupus erythematosus (SLE) sex hormones have been studied before. In RA several studies reported low levels of testosterone, often with an increase of testosterone after attaining remission [27-29]. In SLE the results are not conclusive, with some studies showing suppression of testosterone levels and some not [30]. However, both disease courses and treatment strategies are different from AAV.

Finally, SHBG was raised in over 70% of patients, possibly due to raised production of SHBG in the liver in response to low levels of free testosterone. Levels of free testosterone, which are biologically active, decrease due to binding of SHBG and, subsequently, relatively normal total testosterone levels are measured. This could even underestimate the number of patients with androgen deficiency in a patient population in which only total testosterone levels are measured.

Scores of the RAND-36 and the MFI-20 of patients were significantly worse compared to the control population, except for the subscales of mental health (RAND-36), reduced motivation and mental fatigue (MFI-20). This finding is consistent with the studies of Koutantji *et al*. [5] and Basu *et al*. [7], in which the subscale of mental health of the SF-36 was not significantly reduced. Patients with androgen deficiency scored worse on several subscales of both questionnaires; however, this difference was most evident in subscales reflecting physical functioning, including the PCS. In addition, total testosterone was an important predictor for physical functioning, especially in older patients. Total testosterone, age, VDI and CRP were predictors for fatigue. This demonstrates that both testosterone and damage due to disease activity may play a role in fatigue. In conclusion, this suggests an influence of testosterone on physical performance and fatigue in male patients with AAV.

Our study has several limitations. Fatigue and reduced HRQOL are multifactorial problems and the group of patients is too small to identify all the contributors to these problems. This heterogeneous cohort consisted of patients diagnosed with GPA, MPA and EGPA. With only two EGPA patients, this cohort is probably not representative of patients with EGPA. Additionally, fatigue is also a common complaint among female patients, but they have been excluded from this study, due to the focus on testosterone.

We measured the testosterone levels only once and we did not measure levels of luteinizing hormone (LH) or follicle stimulating hormone (FSH). Therefore, further research is needed to specify if the low testosterone levels are caused by primary testicular failure, by hypopituitary hypogonadism or by a combination of both. Furthermore, we have not evaluated other relevant consequences resulting from low levels of testosterone in this study, in particular sexual dysfunction, which might be more strongly associated with testosterone levels than fatigue is [31].

## Conclusions

This study showed that a substantial number of male patients with AAV in remission have androgen deficiency. Furthermore, we demonstrated that reduced levels of testosterone were associated with reduced physical performance and increased fatigue. Since testosterone is one of the few contributing factors that could be modified, testosterone substitution might be considered in these patients.

We also found evidence that treatment with glucocorticoids might influence androgen production in male patients with AAV. Our findings stress the need for new therapies with less toxic drugs which could not only replace cyclophosphamide but also reduce the need for treatment with glucocorticoids.

Further research should study the effect of testosterone substitution on fatigue, physical performance and health-related quality of life in male patients with AAV.

## List of abbreviations

AAV: ANCA-associated vasculitides; ANCA: anti-neutrophil cytoplasmic antibodies; BVAS: Birmingham Vasculitis Activity Score; CC: creatinine clearance; CRP: C-reactive protein; CY: cyclophosphamide; EGPA: eosinophilic granulomatosis with polyangiitis; ENT: ear, nose and throat; FSH: follicle stimulating hormone; GC: glucocorticoid; GPA: granulomatosis with polyangiitis; HRQOL: health-related quality of life; LH: luteinizing hormone; MCS: mental component summary; MFI-20: Multi-dimensional Fatigue Inventory; MPA: microscopic polyangiitis; MPO: myeloperoxidase; PCS: physical component summary; PR-3: proteinase 3; RA: rheumatoid arthritis; SHBG: sex hormone-binding globulin; SLE: systemic lupus erythematosus; UMCG: University Medical Center Groningen; VDI: Vasculitis Damage Index.

## Competing interests

The authors declare that they have no competing interests.

## Authors' contributions

JT, BB, JS, CS and AB all contributed to the conception and design of the study. JT and BB collected the data necessary for this study and JT, BB, JS, CS and AB made substantial contributions to the analyses and interpretation of the data. JT, BB, JS, CS and AB contributed to the drafting and revising of the content of the manuscript. All authors read and approved the final manuscript.

## Supplementary Material

Additional file 1 **Table S1**. Scores of the RAND-36 and MFI-20 questionnaires. Click here for file
