# Supplementary material for: Androgen deficiency in male patients diagnosed with ANCA-associated vasculitis: a cause of fatigue and reduced health-related quality of life?
Source: Arthritis Res Ther. 2013 Sep 12;15(5):R117. doi: 10.1186/ar4297 (PMC3979147; doi:10.1186/ar4297)
Supplement: Additional file 1 — Table S1. Scores of the RAND-36 and MFI-20 questionnaires. [file ar4297-S1.DOC]

Additional file 1, Table S1. Scores of the RAND-36 and MFI-20 questionnaires.

|  | **All patients (n= 70)** | **Reference** |  | **Normal androgen level (n= 37)** | **Androgen deficiency (n= 33)** |  |
| --- | --- | --- | --- | --- | --- | --- |
|  | Mean (SD) | Mean (SD) | *p* | Mean (SD) | Mean (SD) | *p* |
| RAND-36 |  |  |  |  |  |  |
| General health  perception | 43.0 (21.1) | 72.7 (22.7) | <0.001 | 48.1 (21.2) ^¶^ | 37.2 (19.8)^¶^ | 0.030 |
| Physical functioning | 67.3 (26.3) | 81.9 (23.2) | <0.001 | 76.5 (22.6) | 57.1 (26.5)^¶^ | 0.002 |
| Social functioning | 71.9 (23.3) | 86.9 (20.5) | <0.001 | 77.0 (22.9)^†^ | 66.2 (22.7)^¶^ | 0.052 |
| Role limitations  physical problems | 50.7 (44.4) | 79.4 (35.5) | <0.001 | 59.5 (44.6)^†^ | 40.9 (42.8)^¶^ | 0.081 |
| Role limitations  emotional problems | 71.2 (39.7) | 84.1 (32.3) | 0.008 | 74.3 (36.1) | 67.7 (43.7)^†^ | 0.489 |
| Pain | 71.0 (26.5) | 79.5 (25.6) | 0.009 | 74.1 (27.3) | 67.4 (25.4)^†^ | 0.294 |
| Vitality | 55.1 (20.2) | 67.4 (19.9) | <0.001 | 58.2 (22.7)^†^ | 51.7 (16.6)^¶^ | 0.176 |
| Mental health | 75.0 (15.8) | 76.8 (18.4) | 0.351 | 76.8 (15.1) | 73.1 (16.6) | 0.336 |
| PCS | 41.4 (11.4) | 50 (10) | <0.001 | 44.7 (11.1) | 37.8 (10.9) | 0.012 |
| MCS | 47.8 (9.5) | 50 (10) | 0.055 | 48.3 (9.0) | 47.2 (10.2) | 0.650 |
| MFI-20 |  |  |  |  |  |  |
| General fatigue | 11.93 (4.8) | 9.91 (5.2) | 0.001 | 11.0 (5.2) | 13.0 (4.3) ^¶^ | 0.081 |
| Physical fatigue | 11.81 (4.6) | 8.79 (4.9) | <0.001 | 10.8 (4.9)^†^ | 12.9 (4.1) ^¶^ | 0.062 |
| Reduced activity | 10.69 (4.6) | 8.69 (4.6) | 0.001 | 9.1 (4.2) | 12.5 (4.6) ^¶^ | 0.002 |
| Reduced motivation | 8.93 (3.9) | 8.23 (4.0) | 0.141 | 8.1 (3.3) | 9.8 (4.4)^†^ | 0.064 |
| Mental fatigue | 8.77 (4.5) | 8.33 (4.8) | 0.414 | 8.9 (4.9) | 8.7 (4.0) | 0.855 |

Scores of all patients and references have been compared and scores of patients with androgen deficiency and normal androgen levels have been compared. Scores of patients with androgen deficiency and patients with normal androgen levels have also been compared to the reference population in which a significant difference is demonstrated by the following superscripts; ^†^ significantly worse than reference population (*p* <0.05); ^‡^ significantly worse than reference population (*p*<0.010); ^¶^ significantly worse than reference population (*p*<0.001).
